# Pitfalls in optical on-line monitoring for high-throughput screening of microbial systems

**DOI:** 10.1186/1475-2859-13-53

**Published:** 2014-04-11

**Authors:** Martin Kunze, Simon Roth, Esther Gartz, Jochen Büchs

**Affiliations:** 1 AVT-Chair for Biochemical Engineering, RWTH Aachen University, Worringerweg 1, 52074 Aachen, Germany; 2 AVT-Chair for Enzyme Process Technology, RWTH Aachen University, Worringerweg 1, 52074 Aachen, Germany; 3 Current address: Sandoz, Biochemiestrasse 10, 6250 Kundl, Austria

**Keywords:** High-throughput screening, On-line monitoring, Fluorescent protein, Microtiter plate, Optical measurement

## Abstract

**Background:**

New high-throughput screening systems for microbial systems, e.g. the BioLector technology, are simple to handle and offer various options of optical online measurements. The parallelization and small scale in microtiter plates allow economical high throughput and, hence, to screen many parameters in reasonable time. Fluorescent proteins as fluorescent tags made the tracking of cellular proteins *in-vivo* a routine task. All these tools significantly contribute to the understanding of bioprocesses. But, there are some pitfalls which might mislead the user of such techniques.

**Results:**

In this work the bacterium *E. coli* and the yeast *K. lactis* expressing the recombinant fluorescent proteins GFP, YFP, FbFP and mCherry were investigated. Cultivations were performed applying special microtiter plates with optodes for dissolved oxygen tension (DOT) and pH measurement in the BioLector system. In this way, microbial growth, protein formation, DOT and pH were monitored on-line via optical signals. During these studies it became obvious that fluorescent proteins can interfere with the optical signals leading to incorrect results. In this work these effects are characterized in detail and possibilities are presented how such adverse effects can be corrected or minimized by mathematical procedures or modification of the measuring method. Additionally, it is shown that morphological changes of cells can affect the biomass on-line monitoring via scattered light.

**Conclusions:**

The here reported phenomena refer to typical experiments in biotechnological labs. For this reason these aspects are highlighted in this work to make operators of such valuable techniques as the BioLector aware for potential pitfalls and resulting misinterpretations. With the right approach it is possible to minimize existing problems and deal with them.

## Background

In the field of biotechnology the demand for process development tools is continuously growing. Thereby, the understanding of biotechnological processes is of paramount importance for their development and operation. To face this challenge, the establishment of high-throughput screening techniques became a trend in biotechnology [1,2]. In this context reactor miniaturization was essential to gain the required degree of experimental throughput. Consequently, miniature stirred bioreactors were developed. Different concepts were presented by various groups over the last years [3-9].

In parallel, microtiter plates (MTP) as shaken reaction systems for microbial and enzymatic reactions became more popular in the last years [10]. To perform these reactions under defined conditions different types of MTP were characterized regarding their mass transfer and hydrodynamic properties [11]. In this way factors such as oxygen transfer [12-15] and mixing [16,17] were determined. To improve these parameters even new MTPs were developed. The so called Flower Plate was designed to solve the typical problem of oxygen limitation in MTPs by introducing a baffle like structure to each well of the MTP [18]. In this way limitations, especially in mass transfer, could be overcome.

Fluorescent proteins (FPs) are used as versatile in vivo reporters to study gene regulation and protein synthesis, folding, localization, and activity in bacteria and yeast [19-23]. The most widely used FP is the green fluorescent protein (GFP) and its derivatives. By targeted modification of GFP, fluorescence markers have been developed that span the visible spectrum from deep blue to deep red. But one disadvantage of these proteins is their dependence on oxygen for maturing in order to display fluorescence. To face this problem, an alternative family of fluorescent proteins has been developed, that binds flavin mononucleotide (FMN) as a chromophore [24,25]. Nowadays, further oxygen independent FPs are available [26].

The combination of MTPs and optical measuring techniques enables the high-throughput monitoring of process relevant parameters during cultivations. The so called BioLector technology allows a continuous and non-invasive on-line monitoring of microbial growth and fluorescence signals without interruption of the shaking process and, hence, the gas–liquid mass transfer [27]. Furthermore, dissolved oxygen tension (DOT) and pH value can be determined via special fluorescent dyes immobilized in sensor spots on the MTP bottom [14,28,29]. For both parameters the dual lifetime referencing (DLR) method ensures very high accuracy [30-32]. Taking all together, MTPs as miniature bioreactors can compete with conventional stirred tank reactors regarding their data output. It was also shown that scale-up between the mentioned systems is well possible [33]. Additional to the higher throughput, the easy handling of MTPs makes them perfect for lab automation [3]. The integration of a high-throughput on-line monitoring system, such as the BioLector, to an automated liquid handling robot creates a screening system combining high-throughput experimentation with high information content [34].

In this work we report about typical experiments performed in numerous biotechnological labs, namely the expression of recombinant fluorescent proteins in different host organisms. Important process parameters such as microbial growth, protein formation, DOT and pH value were monitored on-line via optical measurement techniques. During these experiments unexpected pitfalls were identified which might lead to incorrect data about the process. Cultivation results from the bacterium *Escherichia coli* and the yeast *Kluyveromyces lactis*, expressing different FPs, are presented in order to show how fluorescent proteins can influence the optical measuring signals for DOT and pH as well as the scattered light signal for biomass on-line monitoring. Therefore, different proteins fluorescing at different colors in the visible light spectrum (blue, green, yellow, red) were investigated. Subsequently, methods were found to deal with these interferences. Mathematical correction procedures as well as the modification of the optical measurement technique were performed in order to minimize or avoid the interactions. Furthermore, it was shown that the scattered light signal as on-line biomass indicator is sensitive to morphological changes of the cells. In this work we give an overview of potential sources of errors which may occur in biotechnological experiments if optical methods for the on-line monitoring of bioprocesses are applied. Solution strategies are presented where possible.

## Methods

### Microorganisms

The applied microorganisms with their respective vectors for recombinant protein expression as well as their selection markers can be taken from Table 1.

**Table 1:** Applied microorganisms for the recombinant expression of fluorescent proteins

**Target protein**	**Organism**	**Strain**	**Vector**	**Selection marker**
-	*E. coli*	BL21 (De3)		-
YFP	*E. coli*	BL21 (De3)	pRotHi	Kanamycin
FbFP	*E. coli*	BL21 (De3)	pRotHi	Kanamycin
mCherry	*E. coli*	BL21 (De3)	pRSet	Ampicillin
GFP	*K. lactis*	GG799	pKlac1	-

### Media & cultivation

For *E. coli* pre-cultures terrific broth (TB) medium consisting of 12 g L^−1^ tryptone, 24 g L^−1^ yeast extract, 12.54 g L^−1^ K_2_HPO_4_, 2.31 g L^−1^ KH_2_PO_4_, and 5 g L^−1^ glycerol (all ingredients from Roth, Germany) dissolved in water was used. The pH value was 7.2 ± 0.2 without adjustment. For the main cultivation of *E. coli* a modified Wilms and Reuss medium (henceforth referred as Wilms-MOPS medium) was used [35,36]. It consists of 5 g L^−1^ (NH_4_)_2_SO_4_, 0.5 g L^−1^ NH_4_Cl, 3.0 g L^−1^ K_2_HPO_4_, 2 g L^−1^ Na_2_SO_4_, 0.5 g L^−1^ MgSO_4_ · 7H_2_O, 0.01 g L^−1^ thiamine hydrochloride, 20.9 g L^−1^ 3-(N-morpholino)-propanesulfonic acid (MOPS, 0.2 M), 20 g L^−1^ glucose and 1 mL L^−1^ trace element solution. This trace element solution consists of 1.98 g L^−1^ CaCl_2_ · 2H_2_O, 0.54 g L^−1^ CoCl_2_ · 6H_2_O, 0.48 g L^−1^ CuSO_4_ · 5H_2_O, 41.76 g L^−1^ FeCl_3_ · 6H_2_O, 0.3 g L^−1^ MnSO_4_ · H_2_O, 0.54 g L^−1^ ZnSO_4_ · 7H_2_O, 33.39 g L^−1^ Na_2_EDTA (Titriplex III). The pH was adjusted with 5 M NaOH to a value of 7. In dependency on the clone’s resistance, 50 μg mL^−1^ kanamycin or 100 μg mL^−1^ ampicillin were added to the medium from a 1000-fold concentrated stock solution. Recombinant protein expression was induced by adding 0.1 mM isopropyl-β-D-thiogalactopyranoside (IPTG) to the culture medium after 6 h of cultivation from a 100-fold concentrated stock solution.

For *K. lactis* pre-cultures yeast extract peptone (YP) medium was used, consisting of 10 g L^−1^ yeast extract, 20 g L^−1^ tryptone and 15 g L^−1^ glucose [37]. The main culture was performed in synthetic yeast nitrogen base (YNB) medium. A commercial formulation was used in 2-fold concentration (Fluka/Sigma-Aldrich, Munich, Germany). The list of ingredients can be taken from the company’s user guidelines or from literature [37]. For buffering 0.15 M potassium hydrogen phthalate (C_8_H_5_KO_4_) was added. Additional 20 g L^−1^ galactose served as carbon source and inducer for recombinant protein expression.

For *E. coli* pre-cultivation, 10 mL of TB medium in a 250 ml shake flask were inoculated with 50 μL from a cryoculture, and cultures were grown for 8 h at 350 rpm (shaking diameter 50 mm) and 37°C. *K. lactis* pre-culture conditions were the same aside from being grown in YP medium for 12 h at 30°C.

Main cultures were performed applying the BioLector system which allows high-throughput screening of fermentation processes in micro-scale [27,33]. With this technology relevant process parameters such as microbial growth, formation of fluorescent proteins, DOT and pH value are on-line monitored in shaken microtiter plates without interruption of the shaking process. The used BioLector device was obtained from m2p-labs (Beasweiler, Germany). For cultivation so called Flower Plates (MTP-48-BOH, Lot. 1202, m2p Labs, Germany) were used equipped with optodes for on-line monitoring of DOT and pH value. Wavelengths and gain factors for all optical signals can be seen in Table 2. For scattered light and fluorescence measurement the initial light intensity (I_0_), which is mainly attributed to such factors as the media background or the type of the microtiter plate, was subtracted from the original measured data (I-I_0_). All cultivations were performed in triplicate. Parallel cultures were in excellent agreement. The presented results originate from a representative single culture.

**Table 2:** Optical signals and applied setup for BioLector on-line monitoring

**Optical signal**	**λ**_**ex **_**[nm]**	**λ**_**em **_**[nm]**	**Gain**
Biomass (scattered light)	620	-	20
DOT	505	590	60
pH	485	530	45
YFP fluorescence	510	532	60
FbFP fluorescence	450	492	60
mCherry fluorescence	580	610	60
GFP fluorescence	488	520	100

For the main cultivation of *E. coli*, Wilms-MOPS medium was inoculated from the pre-culture, resulting in an initial OD_600_ of 0.1. The already inoculated medium was then transferred to the wells of the MTP. The cultivation was performed at 37°C, a shaking frequency of 1100 rpm, a shaking diameter of 3 mm and a filling volume of 800 μL per well. The plates were sealed with gas-permeable seals (AB-0718, Thermo Scientific, Dreieich, Germany). The conditions for the *K. lactis* main culture were the same aside from being grown in YNB medium at 30°C.

### Protein expression and purification

For the production of fluorescent proteins *E. coli* was cultivated in 250 mL shake flasks with 10 mL Wilms-MOPS medium inoculated from pre-cultures at an initial OD_600_ of 0.1. Cultivation took place at 37°C, a shaking frequency of 350 rpm, and a shaking diameter of 50 mm. Protein expression was induced by adding 0.1 mM isopropyl-β-D-thiogalactopyranoside (IPTG) to the culture medium after 6 h of cultivation. After 24 h of cultivation, OD_600_ was determined and the cells were harvested by centrifugation in 50 mL Falcon tubes at 4000 rpm. Subsequently, the intracellular fluorescent protein was extracted by using the BugBuster® Protein Extraction Reagent (Novagen®/Merck, Darmstadt, Germany) in accordance with the manufacturer’s guidelines. The resulting supernatant, containing the target protein, was then concentrated in ultrafiltration tubes with an exclusion size of 10 KDa (VIVSPIN 20, Sartorius Stedim BioTech, Göttingen, Germany) at 4000 rpm to approx. one tenth of the original volume and afterwards diluted with the same volume of storage solution containing 10 mM NaCl and 10 mM NaH_2_PO_4_. The purified proteins were stored at 4°C.

### Spectral analysis

Absorption spectra of YFP, FbFP and mCherry were determined on a FP-6300 fluorescence spectrometer (Jasco, Groß-Umstadt, Germany) with 2 mL purified protein solution in a 3.5 mL quartz glass cuvette (Type 101-QS, Precision Cell Quartz SUPRASIL®, Hellma, Müllheim, Germany). The absorption spectra were recorded by scanning from 350 nm to 650 nm.

2D fluorescence spectra were determined on a FluoroMax-4 spectrofluorometer (HORIBA Jobin Yvon, München, Germany) with 2 mL *E. coli* cell suspension in a 3.5 mL quartz glass cuvette (Type 101-QS, Precision Cell Quartz SUPRASIL®, Hellma, Müllheim, Germany). Cell suspensions originated from cultivations for protein expression described earlier. The spectra were recorded by scanning excitation and emission wavelengths from 300 to 750 nm.

### FP in-vitro experiments

For in-vitro experiments purified fluorescent proteins were used. For the detailed investigation of the influence on the DOT signal the particular protein solution (preparation described before) was used to create solutions of different fluorescence intensity. Therefore, the stock solution was diluted with the storage solution containing 10 mM NaCl and 10 mM NaH_2_PO_4_. 800 μL of samples prepared in this way were added to each well of the MTP which was shaken at a frequency of 1100 rpm and a shaking diameter of 3 mm at 37°C. To ensure DOT values of 0 and 100% air saturation, the climate chamber of the BioLector was aerated with nitrogen or pressurized air, respectively. In this way Φ_0_ and Φ_100_ were measured, and the parameter K_SV_ was determined applying Eq. 1. Finally, the calibration curve could be calculated due to the Stern-Volmer relationship (Eq. 1).

(Eq.1) $$\frac{\tau_{0}}{\tau }=\frac{\tan\;\Phi_{0}}{\tan\;\Phi }=1+K_{\mathrm{SV}}\cdot \left[\mathrm{DOT}\right]$$

For the corresponding pH experiments the purified protein’s stock solutions were diluted in non-dyed CertiPUR® ready-to-use buffers with pH values of 4–9 (Merck, Darmstadt, Germany) to get solutions with varied fluorescence intensity and pH value. After adding the protein solution to the buffer, the pH was measured again. In all cases the measured pH did not deviate more than ±0.2 from the particular buffer pH. In this way the sigmoidal calibration curve in the pH range from 4–9 could be determined. For mathematical description the Boltzmann equation (Eq. 2a) was modified for the calculation of the pH value from the measured phase angle Φ (Eq. 2b).

(Eq.2a) $$\Phi =\frac{\Phi_{\min}-\Phi_{\max}}{1+e^{\left(\mathrm{pH}-pH_{0}\right)/\mathrm{dpH}}}+\Phi_{\max}$$

(Eq.2b) $$\mathrm{pH}=\ln\left(\frac{\Phi_{\min}-\Phi_{\max}}{\Phi +\Phi_{\max}}+1\right)\cdot \mathrm{dpH}+pH_{0}$$

For DOT reference measurements alternative sensor spots obtained from Presens Precision Sensing (Regensburg, Germany) were used. Sensor spots with (SP-PSt3-NAU-D3-YOP) and without optical isolation (SP-PSt3-NAU-D3-NOP) were fixed with silicone glue to the well bottoms of a Flower Plate without DOT and pH optodes. According measurements were performed in the BioLector device with the same settings applied for conventional DOT optodes from m2p-labs (Table 2).

For the characterization of the effect of the fluorescent protein mCherry on the scattered light signal for on-line biomass monitoring *E. coli* BL21 (De3) cells without an additional plasmid were used. Cultures were grown at the conditions of *E. coli* pre-cultivation described before, but without the addition of antibiotics, and, finally, the respective OD_600_ was determined. Subsequently, cell suspension, purified mCherry stock solution, and storage solution containing 10 mM NaCl and 10 mM NaH_2_PO_4_ were mixed in such relations that samples resulted with varied mCherry fluorescene intensity but a constant OD_600_ of 4.9. 800 μL of the samples prepared in this way were added to each well of the MTP which was shaken at a frequency of 1100 rpm and a shaking diameter of 3 mm in the BioLector at 37°C.

### Scattered light wavelength scan

For the scattered light wavelength scan *E. coli* BL21 (De3) without an additional vector was used. A cell suspension grown at the conditions of *E. coli* pre-cultivation without the addition of antibiotics was diluted with 0.9% [m/v] NaCl solution to prepare samples with varied OD_600_ of 0.06-17.4. For the scattered light scan 200 μL of these samples were added to each well of a 96well MTP (lumox™ multiwell plate, Greiner Bio-One GmbH, Frickenhausen, Germany) which was shaken at a frequency of 995 rpm and a shaking diameter of 3 mm at 37°C. The measurements were performed on an in-house constructed BioLector device operated with the FluoroMax-4 spectrofluorometer (HORIBA Jobin Yvon, München, Germany) equipped with an Y-shaped optical fiber (UV–VIS, LEONI Fiber Optics GmbH, Neuhaus-Schierschnitz, Germany). Wavelengths of 200–800 nm were tested.

### Offline analytics

For offline biomass quantification the dry cell weight (DCW) and optical density at 600 nm (OD_600_) were measured. For DCW determination 500 μL of cell suspension were centrifuged at 14000 rpm for 10 min in pre-dried tubes of known weight. Subsequently, the supernatant was removed and the pellet was washed by re-suspending it in 1 mL water and centrifuged as described before. The supernatant was removed again and the tubes with the pellets were dried for 72 h at 80°C before they were weighed. OD_600_ was determined via a Genesys 20 photometer (Thermo Scientific, Dreieich, Germany) in 1.5 mL micro cuvettes (PS, Plastibrand, Roth, Karlsruhe, Germany). For values higher than 0.5 the samples were appropriately diluted with 0.9% [m/v] NaCl solution.

Galactose concentration in medium was measured by HPLC analysis. After centrifugation of the samples, the supernatant was filtrated through a membrane with 0.2 μm pore size to remove particles. For the measurement the device UltiMate3000 (Dionex, Germany) was used with an Organic Acid-Resin column (250 × 8 mm, CS-Chromatographie Service, Langerwehe, Germany). The eluent was 5 mM H_3_PO_4_ at a flow rate of 0.6 mL/min and 60°C. Peaks were detected by recording the refractive index (Skodex RI-71, Showa Denko Europe, Germany). For data analysis the software Chromeleon (Dionex, Germany) was applied.

For flow cytometric measurements the Guava EasyCyte Mini Base System (Merck-Millipore, Darmstadt, Germany) was used with a gain factor of 8 and a treshold of 2. For optimal measurement the volumetric cell count should range from 50–500 cells L-1. Samples with higher values were appropriately diluted with 0.9% [m/v] NaCl solution.

## Results & discussion

### Effect of FPs on DOT and pH optode signals

**Figure 1:**
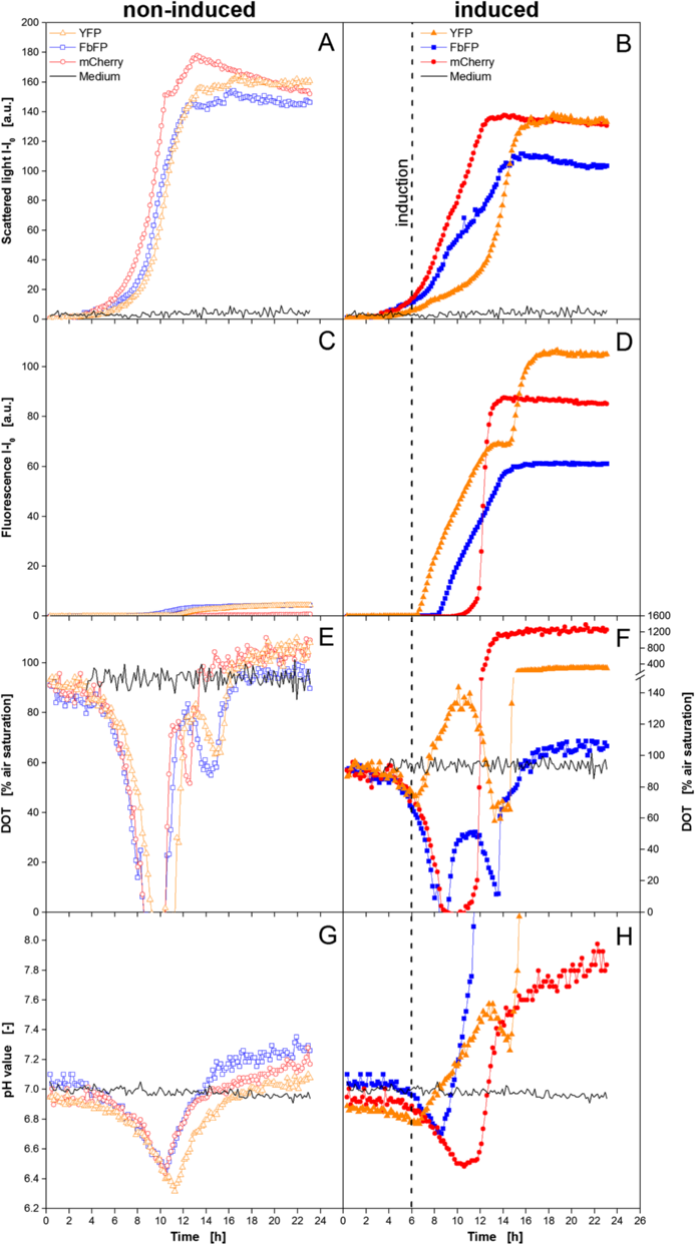
**Cultivation of 3 *E. coli *BL21 clones expressing different fluorescent proteins under non-induced (left column, open symbols) and induced conditions (right column, closed symbols) using the BioLector system.** Online monitoring of microbial growth via scattered light **(A,B)**, fluorescence intensity of the recombinant expressed fluorescent proteins **(C,D)**, DOT **(E,F)** and pH value **(G,H)** via optodes. Note: Altered DOT scale in Figure 1F at higher values. Cultivation conditions: 48well FlowerPlate with optodes for DOT and pH measurement, V_L_ = 800 μL, n = 1100 rpm, d_0_ = 3 mm, 37°C, Wilms-MOPS medium with 20 g/L glucose, induction with 0.1 mM IPTG after 6 h (indicated by dotted line).

To show the influence of fluorescent proteins on the optical on-line monitoring of DOT and pH via optodes, three *E. coli* clones expressing different fluorescent proteins were cultivated under non-induced and induced conditions, applying the BioLector technology. For this investigation three proteins with clearly different spectral properties regarding their excitation and emission wavelength were chosen, namely YFP, FbFP and mCherry, emitting light in the yellow, blue and red light spectrum range, respectively. As a reference pure medium without inoculation was tested, too. The results from these experiments can be seen in Figure 1.

Under non-induced conditions the growth behavior of the three clones is almost the same (Figure 1A). After a *lag* phase of 3 h, cells start growing exponentially. The mCherry expressing clone shows the fastest growth reaching a first maximum after 10.5 h. The clones expressing FbFP and YFP reach this maximum only after 12.5 and 13 h, respectively, thereby showing a slight decrease of the growth rate after 10.5 h. After a short interruption of the exponential growth, all clones have a second increase of the scattered light signal before entering the stationary phase. From earlier experiments it is known that this second growth phase is due to the consumption of the by-product acetate which is a result of oxygen limited conditions and overflow metabolism [35,38,39]. Pure medium shows a constant scattered light signal close to 0 a.u. over the whole cultivation time.

In Figure 1C the production of the fluorescent proteins under non-induced conditions is depicted. As expected, almost no fluorescence was measured for all three clones. Only a small increase could be detected after 9–12 h. These results indicate that leaky expression of the target protein is rather low for these clones in the applied Wilms-MOPS medium.

The DOT curves in Figure 1E go hand in hand with the scattered light signal in Figure 1A. After the *lag* time, where the DOT signal remains at a value of about 90% air saturation, a strong decrease of the DOT occurs as a result of exponential microbial growth. After 9–10 h, the cultivations of all three clones run into an oxygen limitation, indicated by a DOT value of 0% air saturation, which lasts for about 2 h. Besides the formation of acetate, which was mentioned before, this limitation might also be the explanation for the slightly decreased growth rate in the late exponential growth of *E. coli* YFP and FbFP. The strong DOT increase after the oxygen limitation phase indicates the depletion of the major carbon source glucose in the medium. Consequently, no further respiration is possible so that oxygen is recovered in the medium. However, before the cells entered the stationary phase a second drop of the DOT signal is obtained for all three clones. This decrease, accompanied by an increase of the scattered light signal, is caused by the respiration of acetate accumulated during the cultivation [40]. The complete consumption of this by-product after 12.5, 14.5 and 15 h by *E. coli* mCherry, FbFP and YFP, respectively, leads to the final recovery of the DOT to values of 95–105% air saturation. It should be noticed that the DOT level for pure medium over the whole time and also for the cultivations in the beginning and in the stationary phase was not at 100% air saturation as expected. A reason for this might be imprecise calibration parameters given by the manufacturer. Interestingly, *E. coli* YFP and mCherry both ended up at consequently higher DOT values than *E. coli* FbFP and pure medium, which will be explained later.

The curves of the pH values in Figure 1G show the typical behavior of *E. coli* cultivations. Starting at values of 6.9-7.1 the pH steadily decreases after the *lag* phase due to the consumption of ammonia from the medium and the accumulation of acetate. The curves reach their minimum after 10–11 h. This correlates very well with the time when the DOT increased again as a result of glucose depletion. Hence, exponential growth was terminated and, thereby, ammonium consumption and acetate formation stopped. With the subsequent respiration of acetate the pH value increased again. The final values are slightly higher than the initial ones. This is not in agreement with values of 6.6-6.8 obtained from offline samples at the end of the experiment.

Compared to the non-induced conditions, the growth behavior of the three investigated clones differs clearly under induced conditions (Figure 1B). Until a cultivation time of 6 h, the conditions were identical to the experiments without induction. Consequently, no great differences can be obtained in the early phase of the cultivation. After 6 h, 0.1 mM IPTG were added to the medium. From this point, the cultures start varying in their particular growth. Compared to non-induced conditions (Figure 1A), all clones have a distinct lower growth rate after adding IPTG. This is not surprising due to the fact that the overexpression of a recombinant protein can cause an additional metabolic burden to the host organism [38,41,42]. From the curves it is obvious that the perturbed growth occur with some delay and with different strength. While *E. coli* mCherry is poorly affected after 9 h of cultivation, the scattered light signal for the FbFP expressing clone has a much lower slope after this point. The induction effect on *E. coli* YFP is the strongest since it shows the lowest growth rate after already 7 h. It must be considered that these variances between the three clones might also be a result of different cell densities at the time of induction where *E. coli* YFP had the lowest, and mCherry had the highest biomass level. It was reported before that this parameter has a strong influence on the growth and expression behavior of *E. coli*[34]. All clones should have been induced at the same scattered light intensity, but since this was not the focus of this work the more convenient way of a fixed induction time was chosen. What all clones have in common is that the biomass formation recovered over time, so that after 14 h even *E. coli* YFP shows growth rates comparable to these under non-induced conditions. This effect was known before. *E. coli* cells can adapt to the inhibition of recombinant protein expression and recover [38,43]. In the stationary phase a lower final scattered light intensity was obtained for all clones indicating that resources from the medium, which usually would have been used for growth, were redirected to the target protein production.

In Figure 1D it can be clearly seen that significant amounts of the particular fluorescent target proteins were produced by all three clones. Nevertheless, the three curves differ tremendously from each other. The production in *E. coli* YFP starts at 7 h with 1 h delay to the induction and the fluorescence intensity increases steadily for 6 h. Subsequently, a short plateau is formed before the signal increases strongly again after 15 h and reaches its maximum after 17 h. Such a stagnation of the signal followed by a sharp increase is a typical sign for a temporary oxygen limitation. Since YFP is a GFP derivative, it needs oxygen for maturing and generating fluorescence light [24,25]. In the absence of oxygen the protein might be produced but no fluorescence will be detected. When oxygen becomes available, the accumulated non-matured protein matures at once and a high level of fluorescence light is emitted. For *E. coli* YFP this effect occurs when the culture becomes stationary and no oxygen is needed for growth anymore. *E. coli* mCherry shows a first slight increase of the fluorescence intensity after 10 h of cultivation which means 4 h delay to the induction. Subsequently, the signal shoots to the maximum within 2 h. Also this case can be explained by an intermittent oxygen limitation. Compared to the YFP clone *E. coli* mCherry grew relatively fast in the beginning. Consequently, more oxygen is needed for cell growth and maintenance leading to an earlier depletion of the oxygen supply in the medium. In this way no protein could mature before 10 h. Due to the early appearance of oxygen limiting conditions, the precise starting point of the recombinant protein expression cannot be identified. After 9–10 h, the cell's growth rate slightly decreased so that a certain protein amount was able to fluoresce. Two hours later, when the culture became stationary, all the remaining protein matured within short time. Contrary to YFP and mCherry, FbFP’s maturing process is oxygen independent [24,25]. After 8.5 h, *E. coli* FbFP starts producing the recombinant protein. Compared to the YFP clone, the delay after the induction is with 2.5 h longer, but from this point a steady increase of the fluorescence intensity can be observed without any conspicuous behavior. The maximum product concentration is reached simultaneously with the stationary phase. It can be concluded that oxygen independent fluorescent proteins simplify the generation of reliable datasets for product formation kinetics. It should be mentioned that undesired oxygen limitation is not only disadvantageous for maturing of GFP and its derivatives but for bioprocess development in general. Besides misleading fluorescence signals, it can cause inhibited growth and unwanted by product formation which decreases the feasibility of a bioprocess. Consequently, oxygen non-limiting conditions should be ensured even in micro-scale experiments. This can be achieved e.g. by increasing the shaking frequency or decreasing the filling volume per well. Performing cultivations in *fed-batch* mode avoids oxygen limitations, too. Controlled release systems [44], enzyme based *fed-batch* media [45], or microfluidic systems for MTPs [40] are convenient solutions. Nonetheless, this study does not aim for kinetic results so that an adjustment of the conditions was not necessary.

In Figure 1F the corresponding DOT courses are depicted. It can be noticed that to a certain extend they fit qualitatively well to the data of biomass formation and fluorescent protein production. In the beginning, the DOT decreases due to the starting exponential growth. *E. coli* YFP and FbFP show an increase of the DOT after 7 and 9 h, respectively, right at the time when their growth is inhibited by the induction. Simultaneously with the recovery of the microbial growth, the DOT curves start decreasing again before they finally reach the maximum in the stationary phase. *E. coli* mCherry shows a different behavior with a decreasing DOT until a value of 0% air saturation followed by a 2 h lasting oxygen limited phase and a subsequent increase to its maximum. The reason for this course is the much smaller influence of the induction to this clone’s growth and, consequently, a higher oxygen demand over the whole cultivation time. Besides the growth kinetics, this DOT course also approves the assumption that a temporary oxygen limitation is responsible for the late detection of mCherry discussed earlier and the subsequent strong increase of the fluorescence intensity. Surprisingly, no oxygen limitation can be directly observed in the YFP clone’s DOT curve in the time range from 13–15 h which could have caused the plateau and subsequent increase in the YFP fluorescence signal. Completely unexpected are the absolute values delivered by the optical DOT measurement for *E. coli* YFP and mCherry. For the YFP expressing clone DOT values up to 135% air saturation in the growth inhibited phase at 10 h are observed, and almost 400% at the end. For mCherry it is even worse since final values of about 1200% air saturation are reached. For both cultures significant amounts of the fluorescent target protein were detected in parallel to the unrealistic high DOT signals. This fact leads to the assumption that the DOT optodes are strongly influenced by these two proteins. For FbFP no such effect could be observed. Only at the end of the cultivation, the values are slightly higher than those for pure medium.

The pH curves in Figure 1H reveal further surprise. The typical behavior observed under non-induced conditions was not found anymore. The only similarity is a pH decrease in the beginning. After that, *E. coli* mCherry has a slight increase from 10 h on which becomes steeper after 12 h. The culture ends up at a pH of 7.8. The FbFP expressing clone shows the pH increase already after 8.5 h with a high slope and at 11.5 h the signal was out of the measuring range. The YFP clone’s pH value started increasing after 7 h with a short interruption from 13–15 h shortly before leaving the measuring range of pH 9. The final pH values measured offline at the end of the cultivation ranged from 6.7-6.8. So, it becomes obvious again that the unexpected pH courses appear from that time on when the recombinant proteins display their fluorescence. This effect is strongest for FbFP and YFP, but also existent with mCherry.

After the analysis of these experiments, the following points can be concluded:

i) The optical DOT signal is strongly influenced by mCherry fluorescence. Compared to that, the influence of YFP is rather moderate, whereas FbFP has almost no effect.

ii) The optical pH signal is moderately influenced by mCherry, whereas YFP and FbFP seem to have a very strong influence.

The highly parallel experimentation with the BioLector allowed the investigation of several influences and conditions in only one experiment, thereby, saving time and manual effort. From literature, but also from the manufacturer’s information, it is known that the on-line monitoring of DOT and pH via optodes can be prone to certain fluorophores. It is astonishing that the influence of the expressed fluorescent proteins is partly so strong, as the measuring principle is not based on intensity but determined by DLR.

**Figure 2:**
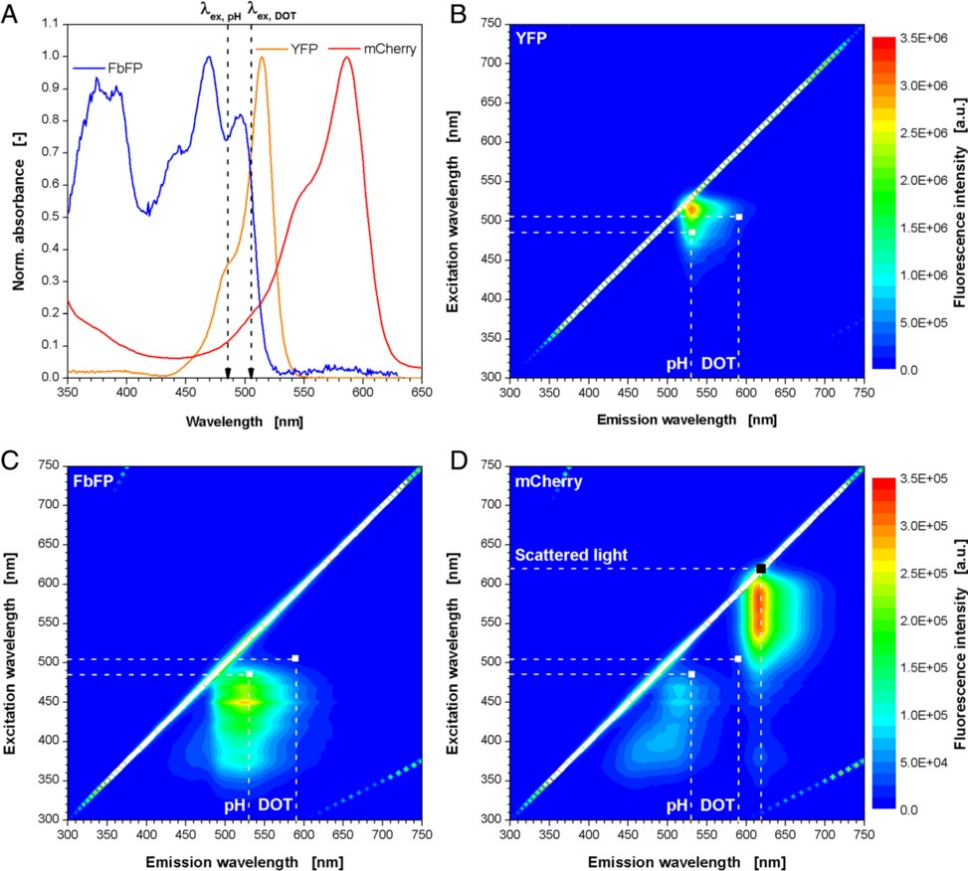
**Absorbance spectra of purified fluorescent proteins (A) and 2D fluorescence spectra of *E. coli *cell suspensions after the expression of the fluorescent proteins YFP (B), FbFP (C) and mCherry (D).** Measuring points for optical monitoring of microbial growth (via scattered light), DOT and pH indicated by dotted lines **(B-D)**. Cultivation conditions: 250 mL shaking flask, V_L_ = 10 mL, n = 350 rpm, d_0_ = 50 mm, 37°C, 24 h, Wilms-MOPS medium with 20 g/L glucose, induction with 0.1 mM IPTG after 6 h. λ_ex,pH_ and λ_ex,DOT_ indicate for excitation wavelengths for optical pH and DOT monitoring. Note: Altered color code for Figure 2B (upper scale) and Figures 2C and D (lower scale).

With a look at the spectra in Figure 2 it becomes clearer why the here tested fluorescent proteins have such an impact on the DOT and pH measuring signals. In the absorbance spectra of the fluorescent proteins it can be seen that all three proteins are able to absorb light energy at the excitation wavelengths for the DOT and pH optode at 505 and 485 nm, respectively (Figure 2A, dotted arrows, λ_ex,DOT_, λ_ex,pH_). This is the prerequisite for fluorescence emission. For influencing the DOT and pH measuring signal there must be emitted fluorescence light in the emission range of the optodes, too. To answer this question, 2D fluorescence spectra of cell suspensions after the expression the particular fluorescent proteins were performed for excitation and emission wavelengths of 300–750 nm (Figures 2B-D). For better visualization the measuring points for DOT and pH are indicated by dotted lines. For YFP (Figure 2B) it can be seen that these points both lie in the fluorescence range of the protein which explains the effect on both signals. The results of the spectrum for FbFP (Figure 2C) also prove the phenomena seen before. Since the measuring point for pH is clearly in the fluorescence range there is an according effect on the on-line signal. On the other hand, the DOT measuring point lies further outside, without any consequences for the measurement. The 2D spectrum of the mCherry expressing culture shows two areas of increased fluorescence (Figure 2D). The DOT measuring point collides with the upper right peak, whereas the pH measuring point lies in the lower left area. Consequently, both signals are prone to interferences with mCherry fluorescence. Surprisingly, the DOT signal is much stronger affected than the pH signal, even though both measuring points are situated in an area of similar mCherry fluorescence intensity. The reason for that is not yet clear.

### Correction of FP’s influences on DOT optode

**Figure 3:**
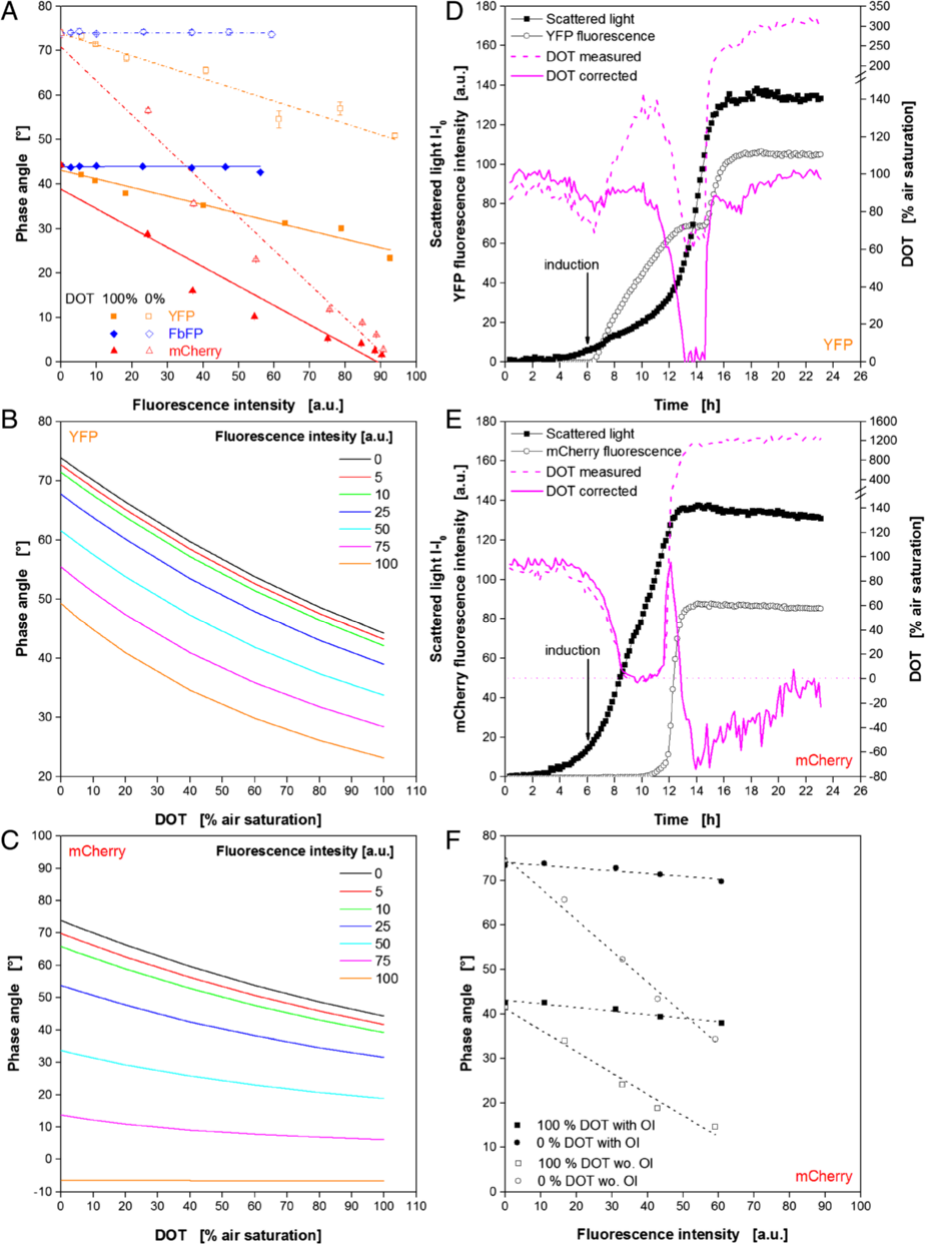
**In-vitro characterization and correction of the influence of different fluorescent proteins on the optical DOT signal. (A)** Dependency of the phase angle as raw signal for DOT monitoring from FbFP, YFP and mCherry fluorescence for DOTs of 100% (closed symbols) and 0% (open symbols) air saturation. **(B)** Change of DOT calibration curve with varied YFP fluorescence intensity. **(C)** Change of DOT calibration curve with varied mCherry fluorescence intensity. **(D)** Correction of the online DOT signal for the cultivation of *E. coli* expressing YFP by using fluorescence dependent calibration curves. **(E)** Correction of the online DOT signal for the cultivation of *E. coli* expressing mCherry by using fluorescence dependent calibration curves. Note: Altered DOT scale in Figures 3D and 3E at higher values. **(F)** Dependency of the phase angle from mCherry fluorescence for optodes with/without optical isolation (OI). Cultivation conditions: 48well FlowerPlate with optodes for DOT and pH measurement, V_L_ = 800 μL, n = 1100 rpm, d_0_ = 3 mm, 37°C, Wilms-MOPS medium with 20 g/L glucose, induction with 0.1 mM IPTG after 6 h (indicated by arrow).

To characterize the interaction between fluorescent proteins and the optical measurement of DOT more in detail, *in-vitro* experiments with purified YFP, FbFP, and mCherry were performed. Therefore, the DOT of solutions with varied fluorescence intensities were determined when aerated with nitrogen or pressurized air. Since no oxygen consumption or formation was apparent in these in-vitro experiments, it can be assumed that the actual DOTs were 0 and 100% air saturation, respectively. In Figure 3A it is shown how the phase angle as raw signal for the DOT changes with increasing fluorescence of the three proteins at 0 and 100% air saturation. As expected from the results before, FbFP has no influence on the DOT signal since it shows a constant phase angle for fluorescence intensities up to 60 a.u. at both DOT values. Contrary to that, the phase angle is clearly dependent on YFP and mCherry fluorescence. In both cases increasing fluorescence intensities lead to decreasing phase angles and, consequently, to misleadingly high DOT measuring values. Interestingly, the relationship between fluorescence intensity and phase angle seems to follow a linear trend. It is also evident that the YFP effect is moderate compared to mCherry. The slope of the decreasing trend lines is lower, and they are almost parallel in the investigated range which means that the influence is similar at DOTs of 0 and 100%. MCherry, on the other hand, shows a steeper slope of both trend lines which are even converging at fluorescence intensities of 80–90 a.u. A reliable measurement at this point is not possible anymore since almost equal phase angles are measured for 0 and 100% air saturation.

Since the relationship of fluorescence intensity and measured phase angle has a linear trend, it was attempted to use this information to correct the DOT signal. For this purpose, the linear functions (Eq. 3a, b) for the trend lines in Figure 3A were used to determine the parameters Φ_0_ and Φ_100_ in dependence on the fluorescence signal.

(Eq.3a) $$\Phi_{0}=m_{0}\cdot \mathrm{FI}+n_{0}$$

(Eq.3b) $$\Phi_{100}=m_{100}\cdot \mathrm{FI}+n_{100}$$

By applying these functions in Eq. 1, K_SV_ can also be described as fluorescence dependent parameter and fluorescence dependent calibration curves can be determined. In Figure 3B exemplary calculated calibration curves are depicted for YFP fluorescence intensities of 0–100 a.u. The curve for 0 a.u. is the actual calibration curve when no fluorescence originating from fluorescent protein occurs. Hence, it should correspond to calibration data given by the MTP manufacturer. Unfortunately, some deviations were obtained during the experiments (data not shown). This finding explains also the slightly too low initial DOT values in Figures 1E and 3F. The supplier’s calibration data delivered initial values of only 90–95% air saturation, whereas a level of 100% was expected. Hence, the new evaluated parameters were used for the further work instead of those provided by the supplier. With increasing protein fluorescence the curves shift more and more to lower values. But, they are parallel to each other, which is beneficial for a signal correction, ensuring a sufficient measuring range also at high fluorescence values. Unfortunately, these relations were not found for mCherry. In Figure 3C it can be seen how the calibration curves change with increasing mCherry fluorescence. It must be noticed that the curves are not parallel and the measuring range becomes narrower so that at 100 a.u. no difference between a DOT of 0% and 100% air saturation can be recognized. Taking a maximum measuring error for the DOT measurement of ±5% into consideration, a critical level is already reached at fluorescence intensities higher than 50 a.u.. Consequently, beyond this point no reliable determination of the DOT from the phase angle is possible anymore.

In Figure 3D the before described method of fluorescence dependent calibration curves was used to correct the DOT signal during the cultivation of *E. coli* YFP under induced conditions (comp. Figure 1). The original signal is indicated by the dotted, the corrected curve by a solid pink line. Additionally, the curves for biomass growth (scattered light) and YFP fluorescence are depicted. It can be noticed that the corrected signal differs strongly from the measured one. Even in the beginning, when no fluorescence was present, the corrected curve is slightly higher. The reason for that is the application of self-determined calibration parameters instead of those given by the MTP supplier. In this way, initial DOT values of almost 100% air saturation are reached as it is expected in the beginning. After 7 h, when the protein fluorescence signal starts increasing, the difference of both curves becomes clearer. The measured DOT signals strongly increased, whereas the corrected signal stayed more or less constant at a level of 90%. This stagnation of the DOT makes sense since the microbial growth is inhibited by the induction at this time as discussed before. After the regeneration of the growth both DOT courses start decreasing again. But, where the original curve forms a plateau at 65%, the corrected signal drops to very low values of almost 0% air saturation, thereby, clearly indicating an oxygen limitation. This correlates very well with the constant fluorescence signal at this time since the lack of oxygen allows no further maturing of the produced YFP. Only after 15 h, when microbial growth came to an end due to the exhaustion of carbon source and oxygen became available again, indicated by a DOT jump, the increase of YFP fluorescence continued. Shortly before the stationary phase from 15.5-17.5 h, the corrected curve shows a decreasing DOT which is not that present in the original data. As discussed before, this is a clear hint for acetate utilization which would have been missed without the signal correction. The biggest effect shows the signal correction at the end, where the realistic and expected value of 100% air saturation is reached instead of more than 300% given by the original measured signal.

In Figure 3E the same procedure was used to adjust the DOT signal during the cultivation of *E. coli* mCherry under induced conditions (comp. Figure 1). Besides the fact that the corrected signal is again slightly higher than the original signal, which was already discussed for the YFP clone, both curves are almost identical up to 12 h. This is not surprising since mCherry fluorescence is hardly detectable in this time. However, the sudden increase of protein fluorescence after 12 h shows the problem for the signal correction. Within very short time the fluorescence signal rises above the critical level, so that no further mathematical correction makes sense. Consequently, even the data correction in Figure 3E doesn’t provide reasonable values at the end of the fermentation.

To summarize these results, it can be stated that it is possible to minimize the influence of fluorophores on the optical DOT monitoring with mathematical methods. Therefore, a mathematical relationship between the disturbing fluorescence and the measuring signal must be identified. Nevertheless, this method has its limits. I our case, the moderate influence of YFP could be eliminated, but it was impossible for the strong influence of mCherry.

Additional to the mathematical solution, another method was tested to minimize the influence of fluorophores on the optode signal. Sensor spots are available which are equipped with a so called optical isolation. Therefore, a black oxygen permeable polymer layer is immobilized on the upper side of the optode directed to the culture broth in order to block disturbing light influences from above. Such optically isolated optodes were attached with silicone glue on the bottom of each MTP well replacing the conventional optodes. In addition, a reference sensor spot without optical isolation was investigated. Since it was not possible to mathematically handle the influence of mCherry on the DOT, this protein was used for the following tests. The results are shown in Figure 3F. The phase angle is depicted in dependency on the fluorescence intensity (comp. Figure 3A). The effect of the optical isolation is astonishing. The sensors spots without optical isolation (open squares) were again prone to the mCherry fluorescence. The values are correlating very well with those of the conventional optodes from m2p Labs (comp. Figure 3A for mCherry). Contrary to that, the sensor spots with optical isolation are almost not affected by the fluorescent protein. Both, the curve for 0% and 100% air saturation, only show a slight decrease of the phase angle with increasing fluorescence intensity. The small remaining error can easily be corrected with the mathematical procedure described before. Consequently, an optical isolation of the optodes towards the culture broth is strongly recommended when interfering fluorophores occur during fermentation experiments. Unfortunately, the optical isolation is not yet available for the Flower Plate and the manual preparation of complete MTPs with isolated sensor spots is highly laborious.

### Correction of FP’s influences on pH optode

**Figure 4:**
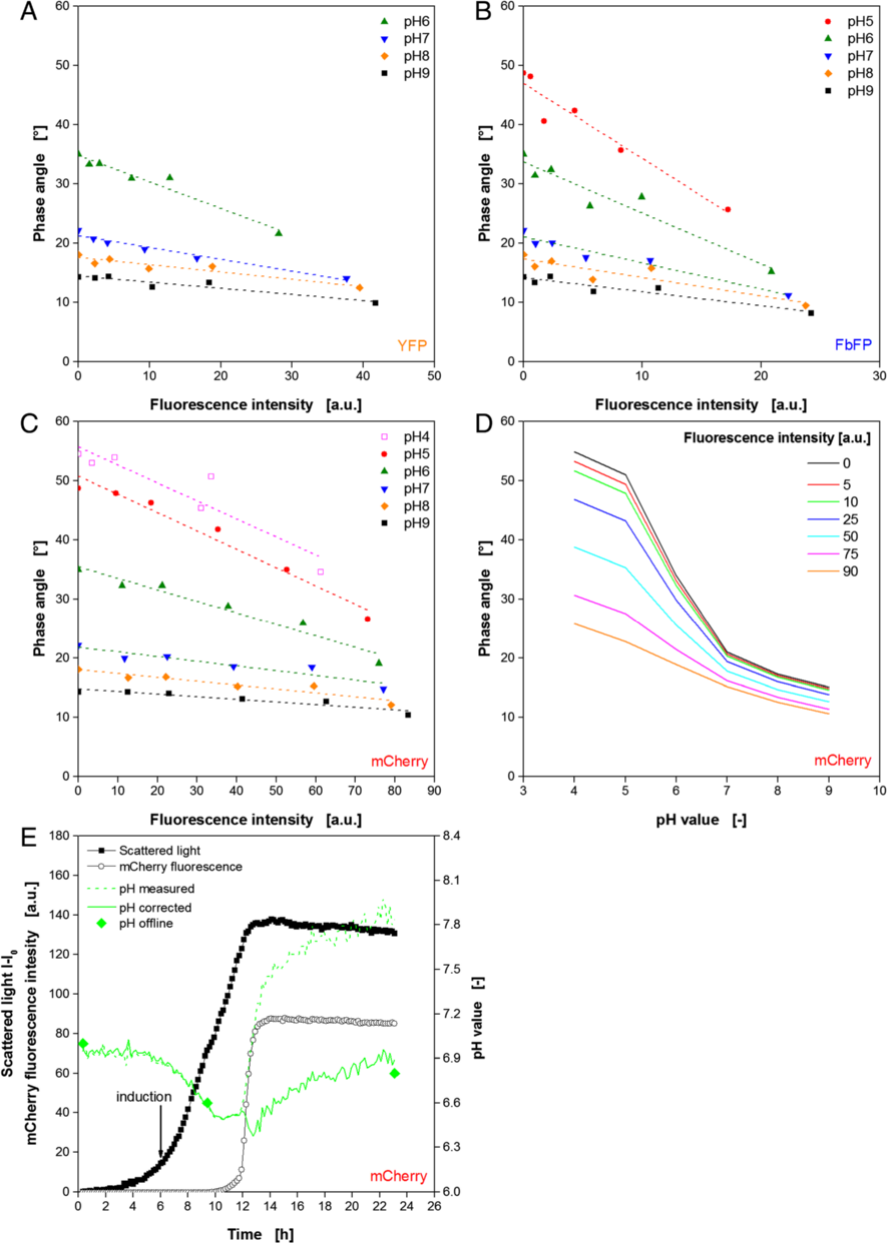
**In-vitro characterization and correction of the influence of different fluorescent proteins on the optical pH signal. (A-C)** Dependency of the phase angle as raw signal for pH monitoring from YFP, FbFP and mCherry fluorescence for different pH values. **(D)** Change of pH calibration curve with varied mCherry fluorescence intensity. **(E)** Correction of the online pH signal for the cultivation of *E. coli* expressing mCherry by using fluorescence dependent calibration curves. Cultivation conditions: 48well FlowerPlate with optodes for DOT and pH measurement, V_L_ = 800 μL, n = 1100 rpm, d_0_ = 3 mm, 37°C, Wilms-MOPS medium with 20 g/L glucose, induction with 0.1 mM IPTG after 6 h (indicated by arrow).

Due to the fact that not only the DOT signal was disturbed by fluorescent proteins, corresponding investigations were performed for the pH measurement. As described before, solutions with varied pH value and fluorescence intensity for the three proteins YFP, FbFP and mCherry were prepared, and subsequently measured with the BioLector. The determined phase angles from the DLR measurement via optodes as raw signal for the pH value can be seen in Figures 4A-C. For all three proteins a decreasing trend is observed with increasing fluorescence. The effect seems to differ dependent on the pH value. Low pH values, represented by higher phase angles, are more prone to the fluorescence than high pH values. As a consequence, the trend lines are converging at certain fluorescence values. For YFP and FbFP these intensities are relatively low at approx. 50 and 30 a.u., respectively. As already observed for the DOT signal before, a reliable measurement of the pH value is not possible beyond these points. And since much higher intensities are reached during the cultivation, namely 105 a.u. for YFP and 60 a.u. for FbFP, a mathematical correction would make no sense for these two proteins. The trend lines for mCherry are converging at a higher fluorescence intensity of approx. 130 a.u., so that a mathematical correction could be successful. By using the linear trend lines in Figure 4C to describe the phase angle as a function of mCherry fluorescence, it is possible to create fluorescence dependent calibration curves. In Figure 4D calculated examples for fluorescence intensities of 0–90 a.u. are depicted. As observed before, the curves move to lower phase angles with increasing fluorescence. More disadvantageous is the resulting measuring range. Without mCherry fluorescence the phase angle reaches over approx. 40° for the pH range of 4–9, whereas at an intensity of 90 a.u. it diminishes to 16°. In this case the pH monitoring becomes more prone to deviations. Nevertheless, this method is applied to correct the pH signal during the cultivation of mCherry. Therefore, the linear equations of the trend lines in Figure 4C are used to determine calibration curves at different fluorescence intensities. The sigmoidal curves in Figure 4D are mathematically described according to Eq. 2b by fitting the parameters Φ_min_, Φ_max_, pH_0_ and dpH applying the Origin data handling software under standard conditions (OriginLab Corp., Northampton, MA, USA). In this way it became obvious that the fitted parameters in Eq. 2b are linear dependent on the fluorescence intensity (data not shown). Subsequently, the resulting linear functions for the parameters were applied in Eq. 2b. For convenience reasons the calculations for the correction were done in MS Excel. Figure 4E shows the results of the procedure. The original signal is indicated by the dotted, the corrected curve by a solid green line. As a reference three samples were taken during the cultivation for offline determination of the pH value (green diamonds). Additionally, the curves for biomass growth (scattered light) and mCherry fluorescence are depicted. It is noticed that up to 12 h the measured and the corrected signal are identical as they show the typical pH decrease in the beginning which was already discussed earlier (comp. Figure 1). This is not surprising since mCherry fluorescence is hardly detectable in this time. The sudden increase of fluorescence after 12 h leads also to a rapid increase of the original pH curve from 6.5 to 7.5. The corrected signal instead is much less affected and increases slowly with the time, as it is expected (comp. Figure 1G). Thereby, the corrected pH values agree very well with the reference offline measurements. Hence, the correction of the pH on-line monitoring was successful.

In summary, it was shown again that it is possible to minimize the effect of a disturbing fluorescence originating from a fluorescent protein also on the pH signal if the influence is not too high. But, it must be mentioned that in this case the detailed characterization of the influence as well as the mathematical steps are more time consuming than in the case of DOT measurement. The method of optical isolation for the pH optode was not tested. But, it reasonable to assume that it will also be beneficial for this application.

**Figure 5:**
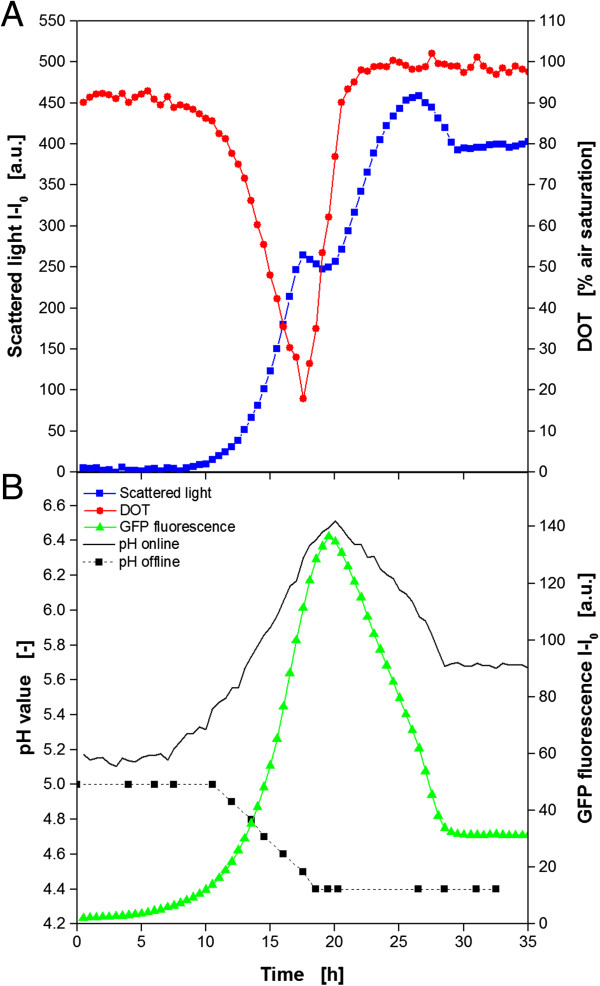
**Cultivation of *K. lactis *GG799 expressing recombinant GFP using the BioLector system.** Online monitoring of microbial growth (via scattered light) and DOT **(A)**, pH value and fluorescence intensity of the recombinant expressed GFP. Additional pH measurement of offline samples **(B)**. Cultivation conditions: 48well FlowerPlate with optodes for DOT and pH online measurement, V_L_ = 800 μL, n = 1100 rpm, d_0_ = 3 mm, 30°C, YNB medium with 20 g/L galactose as substrate and inducer.

In addition to the *E. coli* experiments with YFP, FbFP and mCherry, another cultivation with the yeast *K. lactis* expressing GFP was performed. GFP wasn’t investigated in *E. coli* since the spectral properties of GFP and FbFP regarding excitation and emission wavelength are close to each other (comp. Table 2). Moreover, a GFP expressing *E. coli* clone was not available for this work. It must be considered that the recombinant expression in *K. lactis* is lower compared to *E. coli*. Therefore, a much higher gain factor was used for the detection with the BioLector. Consequently, the fluorescence values are quantitatively not comparable. Figure 5 shows the results of the *K. lactis* cultivation with recombinant GFP expression. In Figure 5A the biomass formation via scattered light is shown in parallel to the corresponding DOT signal. After the *lag* phase of 8 h, the culture starts growing exponentially for 9.5 h. As a consequence, the DOT is decreasing inversely. After 17.5 h, the scattered light signal forms a short plateau and the DOT increases rapidly, indicating the end of the cultivation. Surprisingly, the scattered light shows a second significant increase from 19–26 h. Since the DOT signal already recovered to 100% air saturation in this time, it is hardly conceivable that further biomass formation occurred in this time. The explanation for this phenomenon was subject of further investigations and is discussed later in this work. Figure 5B shows the according results for on-line pH monitoring and GFP fluorescence. As a reference, offline samples were taken continuously for additional pH determination via pH electrode. It can be observed that GFP fluorescence starts increasing from the very beginning. This could be expected since the carbon source galactose, which is also the inducer for GFP production, is already present in the medium at the start. The maximum fluorescence intensity of 135 a.u. is reached after 19 h, and then starts decreasing again. After 29 h, it remains constant at a relatively low level of 33 a.u.. The recombinant protein is obviously degraded right in that time when the second increase of the scattered light signal occurs. The on-line pH signal shows an unexpected behavior, suspiciously similar to the GFP fluorescence. Both signals are increasing, decreasing and stagnating at the same time. Also pH values as high as 6.5 are not typical for yeast cultivations. The offline reference points for the pH substantiate the suspicion that the optical signal is affected by the GFP fluorescence since the offline analysis shows a continuous pH decrease from 5 to 4.4 in the time from 10–18 h. Interestingly, this correlates very well with the first exponential growth phase obtained in Figure 5A, indicating that no further growth occurred after that. Contrary to the on-line pH signal, the optical DOT measurement seems not to be affected by GFP fluorescence since no unexpected values were detected. Consequently, the influence of GFP is similar to that of FbFP with a strong effect on the pH, but no effect on the DOT optode.

### FP’s influence on biomass on-line signal

**Figure 6:**
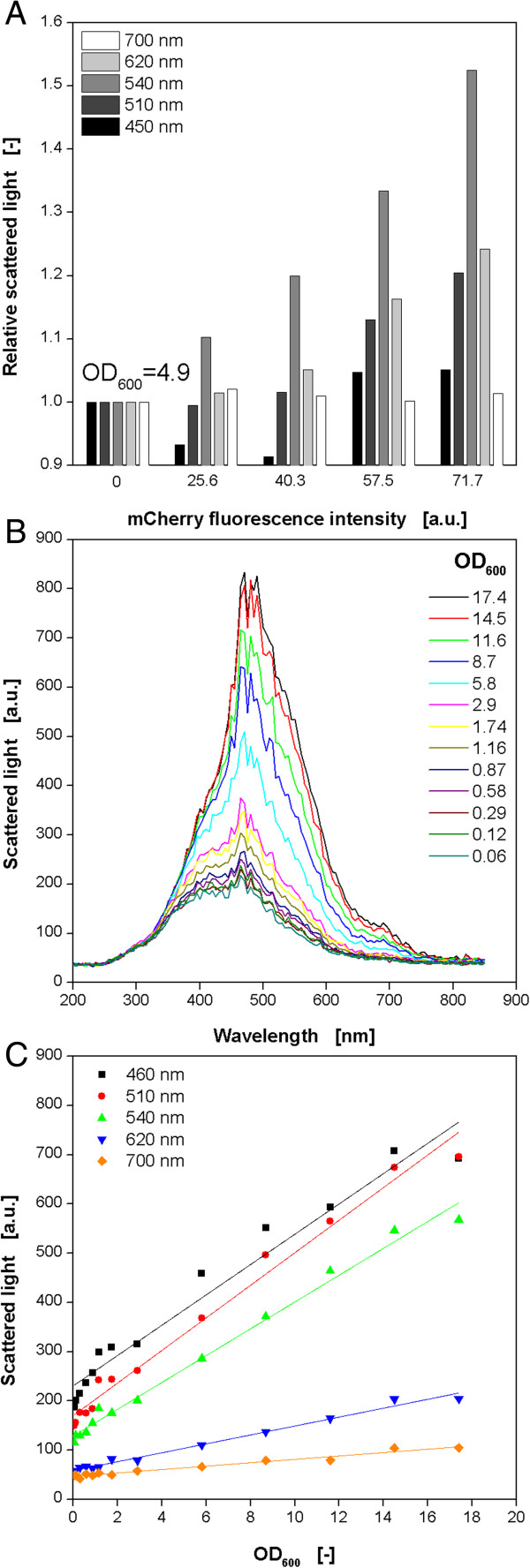
**Characterization of the influence of red fluorescence from mCherry on the scattered light signal for online monitoring of microbial growth. (A)** Dependency of the scattered light signal from mCherry fluorescence at different scattered light wavelengths. Different amounts of purified mCherry resulting in increasing fluorescence intensities were added to a suspension of non-induced *E. coli* cells with an OD_600_ = 4.9. **(B)** Scattered light wavelength scan of *E. coli* cell suspensions with varied biomass concentration (OD_600_). **(C)** Calibration curves between OD_600_ and scattered light intensity resulting from scattered light wavelength scan (comp. Figure 6B) at different scattered light wavelengths.

In a set of experiments with *E. coli* mCherry another problem became obvious. By varying the cultivation conditions different amounts of the FP were produced and the fluorescence intensity varied as well. Surprisingly, the wells with the highest fluorescence intensity showed also the highest scattered light intensity indicating the formation of more biomass (data not shown). This was unexpected since overexpression of recombinant proteins usually causes a metabolic burden to the host, and, therefore, leads to decreased microbial growth (comp. Figure 1A + B). As a consequence, this observation was systematically investigated. Samples were prepared with a constant OD_600_ of 4.9, but varied mCherry fluorescence. From these samples the scattered light intensity was measured in the BioLector (Figure 6A). The conventional scattered light measurement is performed at 620 nm. At this wavelength it can be seen that with increasing mCherry fluorescence intensities also the scattered light signal rises, even though the biomass level is constant in all samples. These results prove the influence of mCherry on the on-line biomass signal. After a look to the 2D fluorescence spectrum of mCherry in Figure 2D, the reason for the effect becomes clear since the measuring point for scattered light (indicated by dotted lines) is close to the peak maximum of the mCherry fluorescence. Consequently, interferences are very likely.

Subsequently, it was tried to find a solution for this problem. The idea was to shift scattered light measurement to another wavelength which is unaffected by fluorescence of mCherry. To see if this is possible, a scattered light wavelength scan of *E. coli* was performed. Therefore, the scattered light intensity of suspensions with varied biomass level (OD_600_ = 0.06-17.4) was measured at wavelengths of 200–850 nm (Figure 2B). In principle, all curves are shaped equally like a triangle with a prominent peak at approx. 460 nm. With increasing OD_600_ the curves move to higher scattered light values. This fact is the prerequisite for biomass quantification. At wavelengths below 320 and above 780 nm almost no differences with varied biomass level occur, a reliable biomass monitoring is not possible in these ranges. For the biomass determination via scattered light a linear relationship of OD_600_ and scattered light intensity would be beneficial. Therefore, the scattered light values for varied optical densities at arbitrarily chosen wavelengths of 460, 510, 540, 620 and 700 nm are pointed out in Figure 6C. For all tested wavelengths it can be recognized that optical density and scattered light are linearly related in the range of OD_600_ values of 2–15. For 620 and 700 nm the linear behavior is also present from 0 on and up to 17.4. The smaller measuring range of the higher wavelength is disadvantageous. At 460 nm it ranges from 200–700 a.u., whereas for 700 nm it’s from 50–100 a.u. Consequently, higher wavelengths are more prone to measuring errors. However, Figure 6A reveals that the measurement at wavelengths of 510 and 540 nm are not independent from mCherry fluorescence. Both signals increase with higher fluorescence intensities. At 450 nm no clear trend can be observed. Contrary to that, the scattered light signal at 700 nm is completely independent of mCherry. Hence, a wavelength higher than 620 nm should be chosen for scattered light measurement in the presence of mCherry fluorescence, but, a compromise must be found ensuring a sufficient measuring range and immunity from interfering fluorescence. Therefore, further wavelengths between 620 and 700 nm should be tested. The 2D spectrum in Figure 2D inspires the assumption that at 640 nm no interference occurs anymore. Another reason for not shifting to lower wavelengths is the fact that also other proteins, e.g. cell or medium components, can display certain fluorescence in this spectral range and, thereby, distort the biomass signal.

To sum this up, it must be recognized that also the on-line monitoring of biomass formation via scattered light measurement at 620 nm is prone to fluorescent proteins, namely mCherry. Similar effects for YFP, FbFP and GFP were not noticed. By shifting the measuring wavelength to values higher than 620 nm, this problem can be avoided. If this is not possible due to instrument limitation, alternative reporter proteins should be used. Additionally, a mathematical correction as performed for DOT and pH monitoring could be possible. This approach was not further investigated in this work since a wavelength shift is much more convenient.

### Effect of morphological changes on biomass on-line signal

**Figure 7:**
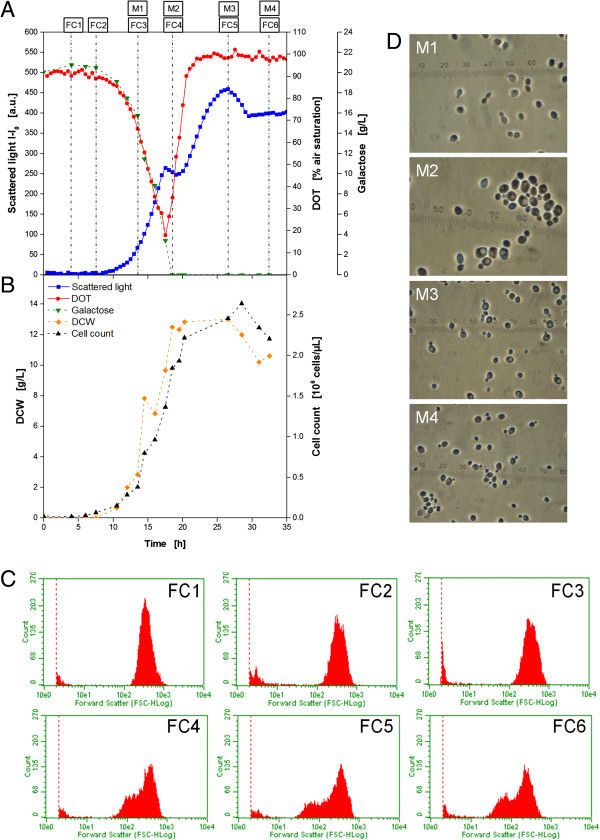
**Cultivation of *K. lactis *GG799 for the investigation of the influence of morphological changes on the scattered light signal for online monitoring of microbial growth. (A)** Online measurement of microbial growth (via scattered light) and DOT (via optodes); determination of galactose concentration in the medium from offline samples (via HPLC). Boxes FC1-6 and M1-4 indicate time points of samples for flow cytometric **(C)** and microscopic **(D)** analysis, respectively. **(B)** Determination of DCW and cell count (via flow cytometry) from offline samples. **(C)** Forward scatter histograms from flow cytometric analysis at different time points (FC1-6). **(D)** Microscope images of yeast cells at different time points (M1-4). Cultivation conditions: 48well FlowerPlate with optodes for DOT and pH measurement, V_L_ = 800 μL, n = 1100 rpm, d_0_ = 3 mm, 30°C,YNB medium with 20 g/L galactose as substrate and inducer.

The results in Figure 5 revealed an unusual behavior of the scattered light signal showing an unexpected second increase of the biomass signal during the cultivation of the yeast *K. lactis*. The signals for DOT and pH value provide no explanation for this phenomenon. The DOT has already returned to 100% air saturation (Figure 7A), and also the pH value remains constant at 4.4 (Figure 5B). Both facts indicate no further growth of the yeast. For further investigation offline samples were analyzed by HPLC for the determination of the galactose concentration which is the carbon source of the medium (Figure 7A). After the *lag* phase, galactose is consumed in parallel to exponential increase of the scattered light. After 18 h, the substrate is depleted. *K. lactis* cells are known to produce ethanol which can be used as carbon source later on. However, HPLC analysis of the samples revealed no further components which could have been used for further microbial growth. Offline samples were analyzed for the determination of dry cell weight (DCW) and cell number via flow cytometry (Figure 7B). The DCW increases exponentially from 8–18 h up to 12.5 g L^−1^. The cell count shows a similar course reaching 2.25 · 10^6^ cells at 20 h. After this time, no significant increase, comparable to that of the scattered light signal, is obtained. All these findings prove the fact that the described phenomenon is not due to microbial growth.

The optical scattered light signal depends on different factors [46]. For the on-line monitoring of biomass concentration the size, surface structure, or granularity of the cells are important parameters. Usually, it is assumed that these parameters do not change significantly during fermentation. To prove this assumption, flow cytometry measurements were performed (Figure 7C). The forward scatter is mainly attributed to cell size. It can be seen that in the first samples taken after 4, 7, and 14 h (FC1-3) only one population occurs ranging from 10^2^-10^3^ a.u.. Interestingly, in the following samples a second population of lower cell size (indicated by lower forward scatter intensity) is revealed (FC4-6). Microscopic images of samples from the cultivation were analyzed in parallel (Figure 7D). At 14 h typically sized yeast cells during the exponential growth are obtained. In the early stationary phase after 18 h, there are still relatively large cells present containing vacuoles. But, in the images M3 and M4 more and more small cells occur. This fact correlates very well with the results from flow cytometry. Finally, it must be concluded that these changes in cell morphology after the depletion of the carbon source galactose are responsible for the unusual behavior of the scattered light signal shown in Figure 7A. Interestingly, this phenomenon occurred only in the synthetic medium YNB, but not in a rich YPD medium (data not shown).

A similar behavior of the scattered light signal was described before for the yeast *Hansenula polymorpha*[47]. It was also reported that shifts in the subpopulations of the light scatter are often associated with events in the cell division cycle [48]. Measurements on single cell level implied this effect to be associated with a change in morphology and heterogeneity in the cell cycle. Unfortunately, this influence cannot be quantified which makes a correction unfeasible.

## Conclusion

New shaken bioreactor systems like the BioLector are simple to handle, and offer various options of optical online measurements. Thereby, the high level of parallelization and the small scale of cultures in MTPs allow economical high throughput and, hence, to screen many parameters in reasonable short time. Since the development of FPs as fluorescent tags, the tracking of cellular proteins *in-vivo* became routine. The application of all these tools significantly contributes to the understanding of bioprocesses. Nevertheless, overreliance on experimental results provided by high-throughput screening procedures applying optical on-line monitoring can mislead the ordinary user. In this work it was demonstrated how fluorescent proteins can influence the optical signals indicating the DOT and pH value. It was shown that:

•YFP has a moderate effect on the DOT optode, the pH signal is strongly affected.

•FbFP has no effect on the DOT optode, the pH signal is strongly affected.

•MCherry has a strong effect on the DOT optode, the pH signal is moderately affected.

•GFP has no effect on the DOT optode, the pH signal is strongly affected.

With a mathematical correction procedure it was possible to minimize the moderate influences, but strong influences were not correctable in this way. By using sensor spots (optodes) with optical isolation even the very strong influence of mCherry on the DOT signal could be minimized. Consequently, an optical isolation is recommended for all measurements. A further solution might be the use of alternative fluorescent proteins in the future. There are reports about variants with fluorescence in the UV spectrum [49], but also in the NIR range [50]. On the other hand, new fluorescent dyes for pH and DOT sensing became available emitting light in the NIR range [51,52]. In both ways interferences of optode and protein fluorescence are excluded.

The scattered light signal as indicator for biomass concentration was proved to be prone to different influences, too. On the one hand, mCherry fluorescence leads to increased scattered light values even though the biomass level is constant. By shifting the measuring point for scattered light to wavelengths higher than 620 nm this effect can be avoided. On the other hand, it was observed that morphological changes of cells can cause unexpected scattered light changes. Unfortunately, this effect is hard to quantify and, hence, not correctable.

In summary, it should be noticed that the phenomena reported here refer to typical experiments in biotechnological labs. For this reason these aspects are highlighted in this work to make operators of such valuable systems as the BioLector aware of potential pitfalls and resulting misinterpretations. With the right methods it is possible to uncover existing problems and correct them.

## Nomenclature

### Abbreviations

DLR Dual lifetime referencing

*E. coli* FbFP *Escherichia coli* BL21 (De3) pRotHi-FbFP

*E. coli* mCherry *Escherichia coli* BL21 (De3) pRSet-mCherry

*E. coli* YFP *Escherichia coli* BL21 (De3) pRotHi-YFP

FbFP FMN-binding fluorescent protein

FMN Flavin mononucleotide

FP Fluorescent protein

GFP Green fluorescent protein

MTP Microtiter plate

YFP Yellow fluorescent protein

YNB Yeast nitrogen base (medium)

YPD Yeast extract peptone D-glucose (medium).

### Symbols

DOT Dissolved oxygen tension [% air saturation]

dpH Step size of sigmoidal pH calibration function [−]

d_0_ Shaking diameter [mm]

FI Fluorescence intensity [a.u.]

I Measured signal intensity [a.u.]

I_0_ Initial signal intensity [a.u.]

K_SV_ Stern-Volmer constant [−]

m_0_ Slope of linear relation between Φ_0_ and FI [° a.u.^−1^]

m_100_ Slope of linear relation between Φ_100_ and FI [° a.u.^−1^]

n_0_ Offset of linear relation between Φ_0_ and FI [°]

n_100_ Offset of linear relation between Φ_100_ and FI [°]

pH_0_ Central pH of sigmoidal pH calibration function [−]

Φ Phase angle (from DLR measurement) [°]

Φ_0_ Φ at DOT = 0% air sturation [°]

Φ_100_ Φ at DOT = 100% air sturation [°]

Φ_max_ Final Φ of sigmoidal pH calibration function [°]

Φ_min_ Initial Φ of sigmoidal pH calibration function [°]

λ_ex,DOT_ Excitation wavelength for DOT optode [nm]

λ_ex,pH_ Excitation wavelength for pH optode [nm]

τ Decay time [ms]

τ_0_ Decay time at DOT = 0% air saturation [ms]

## Competing interests

The authors declare that they have no competing interests.

## Authors’ contributions

MK made the conceptual design of the study and the experimental setup and methods, performed experiments and prepared the manuscript. SR performed the *K. lactis* cultivation experiments including complete analysis. EG designed the experimental setup and methods for the scattered light wavelength scan and performed the experiments. JB assisted with study’s conception, data interpretation and manuscript preparation. All authors read and approved the final manuscript.
